# Impact of dental orientation given to mothers during pregnancy on oral health of their children

**DOI:** 10.1590/S1679-45082016AO3616

**Published:** 2016

**Authors:** Lilian Rigo, Jaqueline Dalazen, Raíssa Rigo Garbin

**Affiliations:** 1 Faculdade Meridional, Passo Fundo, RS, Brazil.; 2 Universidade de Passo Fundo, Passo Fundo, RS, Brazil.

**Keywords:** Oral hygiene, Perception, Oral health, Pregnancy

## Abstract

**Objective:**

To analyze the perception of mothers about oral health of their children, as well as to check the influence of demographic variables, perception and preventive practice in oral health of mothers regarding guidance received during pregnancy.

**Methods:**

Quantitative and cross-sectional field study, with a non-probability sample formed by all mothers who attended the primary healthcare unit of Ijuí (RS), Brazil, from January to July 2014, comprising a sample of 79 women. Self-applied questionnaires were given to these mothers. Data analysis was carried out using descriptive and inferential statistics, the χ^2^ test at a significance level of 5%.

**Results:**

The mothers who received dental orientation during pregnancy had greater perception of oral health of their children. The mean age of mothers was 26 years, most of them attended high school education (32.9%) and worked outside the home (60.8%). There was a statistically significant relation between the outcome variable, dental orientation during pregnancy, and the independent variables: schooling level of mothers, occupation, baby’s first visit to the dentist, duration of breastfeeding, beginning of baby’s tooth brushing and knowledge about dental decay (p<0.005).

**Conclusion:**

Mothers with higher schooling levels and who worked outside the home had more knowledge about oral care, because they received dental orientation during pregnancy. The dental guidance during pregnancy influences the mother in the procedures adopted with their children, as to early oral hygiene, first dentist appointment, duration of breastfeeding, knowledge about the factors that lead to dental decay.

## RESUMO

**Objetivo:**

Analisar a percepção das mães em relação à saúde bucal de seus filhos, bem como verificar a influência das variáveis demográficas, de percepção e prática preventiva em saúde bucal das mães, na orientação odontológica recebida durante a gestação.

**Métodos:**

Pesquisa de campo com abordagem quantitativa e delineamento transversal. A amostragem foi não probabilística com todas as mães que frequentaram o posto de saúde do município de Ijuí (RS), de janeiro a julho de 2014, compondo uma amostra de 79 mulheres. Foram distribuídos questionários autoaplicativos às mães participantes da pesquisa. A análise dos dados foi feita através de estatística descritiva e inferencial, pelo teste do χ^2^ com nível de significância de 5%.

**Resultados:**

Mães que receberam orientação odontológica durante a gestação tiveram maior percepção sobre a saúde bucal de seus filhos. A média de idade das mães foi 26 anos, e a maioria delas frequentou o ensino médio completo (32,9%) e trabalhava fora de casa (60,8%). Houve uma relação estatisticamente significativa entre a variável desfecho, orientação odontológica durante a gestação e as variáveis independentes: escolaridade das mães, ocupação, primeira visita do bebê ao dentista, tempo de amamentação natural, início da escovação do bebê e conhecimento sobre a cárie dentária (p<0,005).

**Conclusão:**

As mães com maior nível de escolaridade e que trabalhavam fora de casa tinham mais conhecimento sobre atenção odontológica, pois receberam orientação odontológica na gestação. A orientação odontológica recebida na gestação influencia as mães nos procedimentos adotados com seus filhos, em relação ao início da higienização bucal, primeira consulta ao dentista, tempo de amamentação, o conhecimento sobre os fatores que levam ao aparecimento da cárie dentária.

## INTRODUCTION

In public health, dental orientation to mothers has been increasingly more focused on young children, including pieces of information on intrauterine life, aiming to guarantee future healthy teeth. Early childhood has been pointed out as being the ideal period for introducing good habits and adopting behavioral patterns that will remain deeply ingrained in the child. One risk behavior, related to diet and/or oral hygiene, which is established during the first year of life, tends to be maintained throughout all childhood.^(1)^

Pregnant women should be considered a priority population group for dental care, considering several factors of this phase: (1) they may present with oral alterations typical of the gestational period; (2) they have accumulated needs that can compromise health of the mother and child; (3) they should be targets of health education programs since they multiply attitudes within the family network, influencing in the family’s eating and hygiene habits; (4) they comprise an easy access group, since they systematically go to the healthcare facilities during this period, which is an important facilitator; also, with this, women can be included in programmed periodic programs, and to not focus on them would be to lose a precious opportunity.^(2)^

According to Bijella,^(3)^ education in oral health means the acquisition of knowledge (information) and the development of skills (instruction), favoring a change in an individual’s behavior and attitudes, creating new values that benefit the health of the patient herself and/or of the parents (motivation). One can observe the need for establishing studies based on prior knowledge of the persons responsible for the children/mothers, as to care with one’s own oral health and that of one’s children, in order to formulate viable proposals and adjust the preventive/educational programs to create new values.^(4)^

Both dental caries and periodontal diseases have a dynamic character and develop by an imbalance in the health process, and not due to a fatality in the life of the individual. In this way, such conditions should be diagnosed and prevented as early as possible, that is, during pregnancy and the child’s first years of life.^(5)^

With the advancements of research in the field of cariology and better comprehension of health/disease process dynamics, the need to implement dental care to the mother/child unit became evident, since science has already proven that the transmission of cariogenic microbiota also occurs vertically, and cariogenic streptococci stabilize in the oral cavity during the eruption phase of the first deciduous teeth. As a consequence of the facts covered, several maternal/child care programs were implemented, currently making the importance of early dental care a consolidated and indisputable fact.^(6)^

Care delivered to the child, and consequently, the education and motivation of the parents as to oral health are the most practical, simple, effective, and low-cost ways to carry out public health programs. However, for a long period, care given to children in Brazil was restricted to the school age, with recommendations that parents should take their children to the dentist after the age of three years, since it was believed that it was only then that the child would be able to cooperate. Today, the literature shows us unquestionably that caries does not wait for the “child’s cooperative age”, and that it indistinctly affects children of any socioeconomic bracket and schooling level, with greater or lesser difficulty in access to care and health education.^(1)^

Currently, the area of pediatric dentistry is focused largely on the health of children and pregnant women. Nevertheless, to promote child oral health, the early insertion of those responsible for orientation programs regarding healthy eating and hygiene habits is essential for prevention of oral diseases.^(7)^ Many studies had the objective of identifying the health profile of the population, thus allowing a contribution towards a better development of public health policies directed to children.^(8)^

## OBJECTIVE

To analyze the perception of mothers as to oral health of their children, as well as to verify the influence of demographic variables, perception and preventive practice in oral health of the mothers, in the dental guidance received during pregnancy.

## METHODS

This is a cross-sectional quantitative study. Sampling was non-probabilistic, with all mothers that attend the central primary healthcare unit of the Municipal Health Department, in the city of Ijuí, located in the Northwestern region of the State of Rio Grande do Sul. They agreed to participate in the research from January to July 2014, making up a sample of 79 mothers.

The study was carried out in the city of Ijuí (RS). Its population was 82,833 inhabitants and the city had many hospital resources.^(9)^ The city had five Primary Healthcare Units (UBS - *Unidades Básicas de Saúde*) set up to see the population. The data were collected only at the central unit of the Municipal Health Department, since these place was in an accessible location and structurally had the largest facilities. The unit provided internal medicine and pediatrics care, and a center for dental specialties. Besides the dentists, it had professionals of gynecology, obstetrics, and specialties of medium complexity, including cardiology, ophthalmology, psychology and speech therapy.

Data gathering was performed based on information given by the mothers, by completing a self-applied questionnaire used during the data collection months. All mothers with children aged less than one year were excluded from the study. The project was submitted to the Research Ethics Committee of *Faculdade Meridional* and approved under CAAE: 31581214.8.0000.5319, Official Opinion number 689.475. The mothers participating in the investigation agreed to take part in the study by signing the Informed Consent Form, and the Secretary of Oral Health of the city signed the Location Authorization Form for the performance of the research.

A descriptive and inferential statistical analysis of the data was performed based on the information obtained from the data collection instrument. The χ^2^ test was used, with a 5% level of significance, as well as the Statistical Package for Social Science software, version 18.0.

For the descriptive analysis, all variables were described with relative and absolute values, to report the distribution of frequencies found in the mothers’ answers. For the inferential analysis, the variable “dental orientation during pregnancy” was defined as dependent. This variable is explained according to the orientation given to the pregnant women by the healthcare professionals (physicians and/or dentists) in the form of conversations/oral communication about the type of cariogenic feeding given to infants, and the most appropriate oral hygiene during the first years of life.

The other variables (age group, occupation, schooling level, the child’s first visit to the dentist, prenatal care, breastfeeding, baby bottle harmful to teeth, use of pacifiers, oral hygiene of the child’s, start of tooth brushing, transmissible tooth decay) were independent. The associations were analyzed, verifying which were statistically significant as to the level of determined probability.

## RESULTS

The mothers comprising the sample were aged 15 to 37 years, mean age of 26 years. Most had completed High School (39.2%) and worked outside the home (60.8%). Almost all the sample (93.7%) had received prenatal care during pregnancy, but most had not received dental orientation in this period (63.3%). A large part of the mothers interviewed took their child to the dentist for the first dental visit during the child’s first year of life (64.6%). When asked about breastfeeding, most mothers stated they had exclusively breastfed up to six months of life (81%), and all believed that use of baby bottle was harmful to teeth (100%). As to the use of pacifiers, 65.8% believed they would damage the child’s teeth. Most mothers performed oral hygiene of their children (79.8%), and 72.2% began brushing their children’s teeth when the first tooth appeared. The majority of the mothers knew that dental caries was a transmissible disease (64.6%).

The descriptive results of the variables are shown on table 1.

**Table 1:** Descriptive analysis of the variables

Variables	n (%)
Age range (years)	
1 (15-23)	25 (31.6)
2 (24-26)	27 (34.2)
3 (27-37)	27 (34.2)
Schooling level	
Never studied	10 (12.7)
Incomplete Junior School	12 (15.2)
Complete Junior School	26 (32.9)
Complete High School	31 (39.2)
Occupation	
Housewife	31 (39.2)
Works outside the home	48 (60.8)
Prenatal care	
No	5 (6.3)
Yes	74 (93.7)
Dental orientation during pregnancy	
No	50 (63.3)
Yes	29 (36.7)
Child’s first visit to the dentist	
First year	51 (64.6)
1-2 years	21 (26.6)
After 2 years	3 (3.8)
Does not know	4 (5.1)
Exclusive breastfeeding, months	
4	9 (11.4)
5	6 (7.6)
6	64 (81.0)
Baby bottle may damage the teeth	
No	0 (0)
Yes	79 (100)
Use of pacifier	
No	27 (34.2)
Yes	52 (65.8)
Cleaning of the child’s mouth	
No	16 (20.3)
Yes	63 (79.8)
Started brushing teeth	
First tooth	57 (72.2)
Complete primary dentition	16 (20.3)
Does not know	6 (7.6)
Transmissible tooth decay	
No	15 (19.0)
Yes	51 (64.6)
Does not know	13 (16.5)

The inferential analysis showed the associations between the independent variables and the variable of outcome “orientation during pregnancy”, as shown on table 2.

**Table 2:** Inferential analysis between the dependent variable “orientation during pregnancy” and the independent variables

Variables	Received dental orientation during pregnancy			

	Yes	No	Total	p value
	n (%)	n (%)	n (%)	
Age range, years				0.131
15-23	18 (36)	7 (24.1)	25 (31.6)	
24-26	13 (26)	14 (48.3)	27 (34.2)	
27-37	19 (38)	8 (27.6)	27 (34.2)	
Total	50 (100)	29 (100)	79 (100)	
Schooling				<0.001*
Never studied	10 (20)	0 (0)	10 (12.7)	
Incomplete Junior School	12 (24)	0 (0)	12 (15.2)	
Complete Junior School	26 (52)	0 (0)	26 (32.9)	
Complete High School	2 (4)	29 (100)	31 (39.2)	
Total	50 (100)	29 (100)	79 (100)	
Occupation				<0.001*
Housewife	30 (60)	1 (3.4)	31 (39.2)	
Works outside the home	20 (40)	28 (96.6)	48 (60.8)	
Total	50 (100)	29 (100)	79 (100)	
Prenatal care				0.094
No	5 (10)	0 (0)	5 (6.3)	
Yes	45 (90)	29 (100)	74 (93.7)	
Total	50 (100)	29 (100)	79 (100)	
Child’s first visit to the dentist				<0.001*
First year	22 (44)	29 (100)	51 (64.6)	
1-2	21 (42)	0 (0)	21 (26.6)	
After 2 years	3 (6)	0 (0)	3 (3.8)	
Does not know	4 (8)	0 (0)	4 (5.1)	
Total	50 (100)	29 (100)	79 (100)	
Exclusive breastfeeding, months				0.005*
4	9 (18)	0 (0)	9 (11.4)	
5	6 (12)	0 (0)	6 (7.6)	
6	35 (70)	29 (100)	64 (81)	
Total	50 (100)	29 (100)	79 (100)	
Use of pacifier				0.577
No	17 (34)	10 (34.5)	27 (34.2)	
Yes	33 (66)	19 (65.5)	52 (65.8)	
Total	50 (100)	29 (100)	79 (100)	
Cleaning of child’s mouth				<0.001*
No	16 (32)	0 (0)	16 (20.3)	
Yes	34 (68)	29 (100)	63 (79.7)	
Total	50 (100)	29 (100)	79 (100)	
Started brushing				<0.001*
First tooth	28 (56)	29 (100)	57 (72.2)	
Complete primary dentition	16 (32)	0 (0)	16 (20.3)	
Does not know	6 (12)	0 (0)	6 (7.6)	
Total	50 (100)	29 (100)	79 (100)	
Transmissible tooth decay (caries)				*<0.001
No	13 (26)	2 (6.9)	15 (19)	
Yes	24 (48)	27 (93.1)	51 (64.6)	
Does not know	13 (26)	0 (0)	13 (16.5)	
Total	50 (100)	29 (100)	79 (100)	

*p<0.005. Statistically significant difference.

There was a statistically significant relation between the variable of “outcome” and the schooling level of mothers (p<0.001), identifying that, among mothers with greater time of schooling (complete High School), 100% received dental orientation during pregnancy. There was also a significant relation regarding the variable “outcome” and the variable “occupation” (p<0.001), in which 96.6% of mothers who worked away from home received dental orientation during pregnancy. Another significant association was related to the child’s first visit to the dentist: 100% of mothers who received orientation during pregnancy took their children to a dental visit during the first year of life (p<0.001). As to breastfeeding, there was an influence of this variable with dental orientation during pregnancy, in which 100% of mothers breastfed their children up to six months of age (p=0.05). Concerning the variables “cleaning” and “started brushing” the child’s teeth, both had a significant association with dental orientation during pregnancy, verifying that 100% of mothers cleaned the mouth of their children and initiated with this when the first tooth erupted (p<0.001). The variables related to knowledge about dental caries were also statistically significant (p<0.001), showing that 93.1% of mothers who knew that caries is a transmissible disease were those who had received dental orientation during pregnancy.

## DISCUSSION

Mothers play a fundamental role as messengers of good behavior regarding their children’s oral health. Thus, the greater their knowledge about positive attitudes towards oral habits, the better the children’s oral condition.^(10)^ Implementing prenatal dental care promotion by means of posters and leaflets has brought a great advancement on the national level, with direct reflexes on the behavior of mothers. However, even with these advancements, in this study, we observed that among the mothers interviewed, a minority of them obtained dental orientation during pregnancy, corroborating a similar study in which mothers, when questioned as to whether they had received any orientation related to their children’s oral hygiene care during the prenatal visits, 48% stated that they had never received any type of guidance related to dentistry.^(1)^ In another study, carried out in the city of Caruaru (PE), with pregnant women, most of those interviewed from both groups (private and public), had never received information on dental care of their child; those who had, had received it only from their dentist. Nevertheless, most of the pregnant women reported the need to receive oral health orientation from the complete healthcare team.^(11)^ Such a fact is contrary to the data found in another study, conducted in Campina Grande (PB), in which a small group of mothers interviewed received information on their child’s oral hygiene, and the pediatrician had been the main messenger. Therefore, joint action between the dentistry sector and the pediatrics unit is suggested, affording better care to their pediatric patients.^(12)^

The central unit of the Municipal Health Department of Ijuí (RS), guarantees easy access and quality in prenatal care, offering appropriate care, with frequent medical care to all mothers who seek free treatment. Even so, there is a small percentage of mothers who were not followed up by healthcare professionals during pregnancy.

The analysis of the results of this study showed a statistically significant association between the mothers’ schooling level and the dental orientation received during pregnancy. Such result is in accordance with findings from an investigation carried out with 78 mothers in the city of Bauru (SP), with results showing a significant correlation between the degree of knowledge of mothers about oral health and their education level. There was greater knowledge relative to care of their children among the mothers who had spent more time in school.^(5)^ In a study that evaluated the knowledge of mothers on the oral health of children aged 1 to 4 years, the authors observed that maintenance of the children’s oral health is influenced by knowledge and beliefs of their parents. Mothers with higher education qualification and greater access to information obtained through dentists had better results in knowledge of oral health of their children.^(13)^ This demonstrates the importance of pieces of information passed on to the mothers or persons responsible for the children, so that there is an effective education in oral health, which is essential for maintenance of health and prevention of oral diseases.

Similar to schooling level, mothers who work outside the home have greater access to information and knowledge. In this study, there was also a statistically significant relation in the variable “mother’s occupation”, thus revealing that mothers who work away from home received dental orientation during pregnancy and were better informed than those who worked at home (housewives). Other studies investigated the association between knowledge and the practice of mothers with their children and socioeconomic variables, similar to the variable “occupation” investigated in this study. A survey conducted in a city in the state of Santa Catarina (SC), showed that as the socioeconomic level decreased, the percentage of mothers that did not receive information increased, and the knowledge level was lower.^(14)^ In a study carried out in Manaus (AM), the perception of oral health by the pregnant women was considered low. Additionally, it was determined that in this region, the number of pregnant adolescents with a low schooling level and a family income below one minimum wage is growing.^(15)^ Hence, one can state that socioeconomic conditions directly interfere in factors such as health and education.

There was a significant association in the results of this study relative to the first visit of the child to the dentist and the dental orientation received during pregnancy. The mothers who received orientation took their children to the dentist within the first year of life. This is the most recommended period by dentists for the child to be evaluated, since the mother or person responsible for the child will be informed as to the child’s current oral status, besides receiving information from qualified professional about how to prevent and avoid oral problems in the deciduous and permanent teeth.

As to “breastfeeding”, there was influence of this variable in the dental orientation received during pregnancy, and all mothers of the sample nursed their children to six months of age. In a study performed during prenatal care, 80% of pregnant women, when questioned about duration of breastfeeding, answered that the ideal period is equal to or greater than six months.^(16)^

The variables “oral cleaning” of the child’s mouth and “start of tooth brushing” had a significant association with dental orientation during pregnancy, showing that all mothers cleaned their children’s mouth as from eruption of the first deciduous tooth. These results corroborate those of the study in Campina Grande (PB), where 73.8% of mothers routinely cleaned their child’s mouth; most of these mothers performed oral cleaning with brushing, which is the most used method; and the time when they started oral cleaning was before the eruption of the first deciduous tooth. However, few mothers cleaned their child’s mouth three times a day.^(12)^ Such results are still similar to those of the study that evaluated knowledge and practices of oral health of those responsible for the children seen at a teaching clinic, where it was noted that most of the children began their oral cleaning between six and 12 months of age, and between 1 and 3 years of age they already did their own oral hygiene.^(17)^ A study of the profile of pregnant woman and her perception as to early dental care revealed that oral hygiene of children is practiced by most mothers, but half of the sample never received any type of orientation as to oral hygiene of their child, and did not even know of any type of cleaning mechanism.^(1)^ In another study, conducted at a university hospital during prenatal care, most pregnant women interviewed stated they considered it necessary to do oral cleaning of the newborn’s mouth, but these women had many doubts as to how to perform this cleaning.^(16)^ One can observe that, when healthcare professionals provide mothers orientation in an adequate manner, education and knowledge are effectively absorbed. In a clinical study in which there was intervention of dentists, the frequency of tooth brushing increased after the group of mothers of children aged six to 18 months received ongoing education for a period of eight months.^(10)^ Thus, in this present research, we were able to verify that the mothers interviewed were aware of the need to implement oral hygiene measures for their children as early as possible, in order to avoid future oral complications. The mothers proved to have adequate knowledge as to the care and habits related to oral health.

The variable related to “knowledge about dental caries” was also statistically significant, since the mothers that know that caries is a transmissible disease were those that received dental orientation during pregnancy. This means that the mothers know about a possible transmission of cariogenic bacteria from mother to child. The perception of the pregnant women as to severity of the oral diseases is related to the adoption of care at home and to search for dental care; the latter item, however, only for solving an existing problem. A study performed in Porto Alegre (RS), with persons responsible for the children seen at the clinic of a dentistry college showed that more than half those interviewed revealed some form of knowledge about dental caries, but less than half had never received information about the condition and did not consider it a transmissible disease.^(17)^ In another study, most of the parents showed some knowledge on the eruption of the child’s first tooth, but very little knowledge about oral hygiene and the development of dental caries.^(16)^

Dental caries is considered a public health problem, since the disease affects children during the initial phase of tooth eruption and can be influenced by the habit of nighttime nursing, due to the high consumption of carbohydrates and sugars, and the lack of adequate oral hygiene. Thus, many studies associate the high rate of dental caries in children to the mothers’ lack of knowledge about oral health of their children, low socioeconomic level, and low maternal schooling level.^(6)^ Dental caries is still the most prevalent oral disease in the population, regardless of age, and it has a multifactoral etiology. Besides maternal milk, a study with mothers of children aged from zero to 18 months identified that the mothers used food with an elevated cariogenic effect for their babies, observing that the adoption of healthy eating habits, with an adequate nutritional standard and restricted consumption of high-sugar foods, is a difficult goal to be reached. Therefore, it is suggested that greater attention should be given to the need for early oral hygiene.^(18)^ Positive results are observed when mothers are informed after birth of their children. In a longitudinal study, 480 mothers of children aged 6 to 18 months were randomly selected from a primary healthcare unit, in which the use of the nighttime baby bottle was noted only in the group that did not receive orientations regarding oral health (control group).^(10)^

One of the limitations of this study was that it did not check the dental characteristics of children, in order to analyze the influence of the mothers’ knowledge acquired during pregnancy in the oral clinical characteristics of children. Further studies are necessary to investigate knowledge and perception of mothers, besides the oral clinical examination of their children.

Ample awareness of oral health problems is needed nationwide, to avoid complications caused by negligence and lack of self-care, with the support of healthy lifestyles, by adopting appropriate oral hygiene and eating habits, periodic visits to the dentist, based on an ongoing training of pregnant women and mothers of children during the initial tooth eruption phase. Mothers with regular access to dental care are more inclined to take their children to the dentist and to develop behaviors and habits that promote oral health.^(19)^There is a need for healthcare professionals to engage in the search for strategies to improve the quality of guidance given to the population, making viable the adoption of oral health habits at home and demystifying dental treatment. Considering all these factors, dental treatment might be seen as a fundamental part of integral child health.

A study with pregnant women who received prenatal care at the primary healthcare unit showed a low percentage of them was given orientation regarding oral health during this period, which demonstrates the need for greater integration of the Family Health Strategy team, since this is a priority group due to the role that these women play in promoting oral health at home.^(20)^ The first form of health education for children should be learning from the mother, who should be educated during pregnancy. This should be an ongoing process, regardless of the level of education.^(21)^

Corroborating several studies,^(7,8,15,20-23)^ the data analyzed contribute towards science and society, confirming the need to implement oral health programs in partnership with the prenatal program. The aim is to make them aware and guide them to care for oral health of their children. The preparation of these programs should be developed by the current governments at the three levels, so that the efforts have a permanent and efficient nature for the population. This is due to the fact that current oral health programs give priority to the age group 5 to 12 years, leaving out the phase when the first deciduous teeth erupt, which would involve the infants.

## CONCLUSION

Mothers who received dental orientation during pregnancy had a greater perception of oral health of their children. And those with a higher schooling level and who worked away from home knew more about dental care, since they received dental orientation during pregnancy.

Dental guidance during pregnancy influences the mothers in the procedures adopted with their children, including the beginning of oral hygiene, the first visits to the dentist, duration of breastfeeding, and knowledge about the factors that lead to the appearance of dental caries, improving the perception of oral health of their children.
